# Toxicity mechanisms of aflatoxin M_1_ assisted with molecular docking and the toxicity-limiting role of trans-resveratrol

**DOI:** 10.1038/s41598-022-18791-8

**Published:** 2022-08-25

**Authors:** İlknur Güç, Emine Yalçin, Kültiğin Çavuşoğlu, Ali Acar

**Affiliations:** 1 Department of Biology, Faculty of Science and Art, Giresun University, Giresun, Turkey; 2 Department of Medical Services and Techniques, Vocational School of Health Services, Giresun University, Giresun, Turkey

**Keywords:** Biotechnology, Cell biology, Molecular biology

## Abstract

In this study, AFM_1_ toxicity and the protective role of trans-resveratrol (t-rsv) against this toxicity were investigated with the help of multiple parameters in albino mice. As a result, AFM_1_ (16 mg/kg b.w) administration caused a decrease in body, kidney and liver weights. This reduction was associated with a decrease in feed consumption. AFM_1_ induced an increase in AST and ALT enzyme parameters and BUN, creatinine and MDA levels and a decrease in GSH levels. These increases have been associated with liver and kidney cell damage. AFM_1_ decreased MI and encouraged increases in MN and CAs numbers. The decrease in MI was correlated with AFM1-tubulin and the increase in CAs was associated with the AFM_1_-DNA interaction, which was demonstrated by molecular docking and spectral shifting. Besides, the decrease in DNA damage and amount was demonstrated by the comet assay technique. Administration of t-rsv (10 and 20 mg/kg b.w) reduced the toxic effects of AFM_1_ and caused a dose-dependent improvement in all physiological, biochemical and cytogenetic parameter values studied. For this reason, foods containing t-rsv or food supplements should be consumed in the daily diet to reduce the effect of toxic agents.

## Introduction

Mycotoxins are secondary metabolites produced by fungi and exhibit toxic effects on organisms. In general, mycotoxins are low molecular weight compounds synthesized by filamentous fungi during secondary metabolism, and their chemical structures can vary from simple C_4_ compounds to complex substances. In the agriculture and food industry, there are various mycotoxin groups including aflatoxins, ochratoxins, fumonisins, zearalenone and patulins^1,2^. Aflatoxins are a sub-group of mycotoxins produced by *Aspergillus* species such as *A. flavus* and *A. parasiticus*. Aflatoxins are classified into six different groups depending on the structural diversity. The toxic effects of aflatoxins emerge after their metabolism and biotransformation in organisms. Aflatoxin toxicity generally includes carcinogenic, mutagenic and teratogenic effects. These toxic effects vary according to species diversity, sex, age, nutritional status and the presence of other chemicals. In addition, the dose level of aflatoxin, type and duration of exposure are also very important. Aflatoxin-related toxicity order is Aflatoxin B_1_ > Aflatoxin M_1_ > Aflatoxin G_1_ > Aflatoxin B_2_ > Aflatoxin M_2_ > Aflatoxin G_2_^3,4^. AFB_1_ is the most studied aflatoxin species and its toxic effect is best clarified. Aflatoxins are metabolized by microsomal enzyme systems and various intermediate metabolites are formed as a result of this reaction. These intermediate metabolites include hydroxylated derivatives and highly reactive epoxide metabolites. Aflatoxin M_1_ (AFM_1_) metabolite is formed as a result of hydroxylation of aflatoxin B_1_. AFM_1_ and AFM_2_, symbolized by "M" since they are milk-derived toxins, are formed as a result of biotransformation of aflatoxin B_1_ and B_2_, respectively. AFM_1_ is also produced by *Aspergillus flavus* and *Aspergillus parasiticus*, which pollute plants and plant products^5,6^. AFM_1_ has been detected in milk or dairy products from animals consuming mold-contaminated feeds. The main sources of aflatoxins in feeds are contaminated peanut, meal, corn and cottonseed meal. Some of the AFB_1_ in contaminated feed is converted to AFM_1_ in the liver after ingestion. AFM_1_ combines with glucuronic acid and is excreted through bile or enters the systemic cycle and is either excreted in the urine or passes into the milk^5,7^.

Studies on aflatoxin species have mostly focused on AFB_1_. Studies on AFM_1_ are mostly related to the detection of its presence in milk and dairy products, and toxicity studies are not yet at the desired level. Considering that the consumption of milk and dairy products is essential, especially in children of the developmental age, the investigation of AFM_1_ toxicity has priority compared to other aflatoxins. From this point of view, in this study, a comprehensive toxicity profile of AFM_1_ in albino mice was investigated. In addition, the effects of trans-resveratrol (t-rsv), an antioxidant molecule, in limiting AFM_1_ toxicity were investigated. Resveratrol, which is intensely synthesized by pine trees, legumes and especially grapes, is produced by at least 72 different plant species in the presence of various stress factors. Sources of resveratrol in the human diet are grapes, peanuts and wine. Grape juice and red wine contain high levels of resveratrol. Resveratrol is produced in plants against stress conditions such as thirst, infection, ultraviolet and ozone exposure. Resveratrol consists of two phenolic rings linked by styrene bonds and has two isomers, cis- and trans-. While the two isomers are found together in some plants and wine, grape extract does not contain cis-resveratrol^4,8^. Cis isomerization occurs when the trans-isomer, which is more dominant and stable than the cis isomer, is exposed to sunlight or ultraviolet radiation at a wavelength of 254 or 366 nm. T-rsv, which has many biological activities such as antioxidant, anti-inflammatory, anticarcinogenic, antidiabetic, neuroprotective and photoprotective, is a very promising compound due to its applications in the cosmetic and pharmaceutical industries^9,10^. T-rsv can prevent and slow down oxidative damage in cells with its antioxidant properties. This property is due to the electron-donating property of t-rsv and its oxide reducing ability, which neutralizes reactive oxygen species. The protective properties of t-rsv, which has the ability to eliminate free radicals, O_2_^⋅−^ and H_2_O_2_ activities, have also been reported against oxidative damage and many diseases^2,11^. T-rsv also exhibits a protective role as an activator of sirtuin 1 (SIRT_1_). SIRT_1_ is an NAD^+^ dependent deacetylase that cleaves acetyl groups from other proteins. As a result of the activation of SIRT_1_, cytoprotective effects emerge through many mechanisms such as antiapoptotic, antioxidative, antiinflammation, mitochondrial biogenesis and autophagy. It has been reported in the literature that resveratrol has a protective effect against oxidative damage and toxicity caused by chemicals^12^. However, there is no study in the literature reporting its effect against toxicity caused by AFM_1_. Therefore, with this study, the protective property of t-rsv against AFM_1_ toxicity was reported for the first time in the literature.

The protective ability of t-rsv against multiple toxicities caused by AFM_1_ in albino mice was investigated in a dose-dependent manner. The toxicity profile of AFM_1_ and the protective role of t-rsv were investigated by multiple analyzes such as body weight, organ weight, feed consumption, serum parameters, glutathione (GSH) and malondialdehyde (MDA) levels, mitotic index (MI), micronucleus (MN) and chromosomal abnormalities (CAs) frequencies. The toxicity mechanism has been interpreted by relating all parameters to each other. It is very difficult to explain the mechanism of action of chemicals in cells and to reveal the secrets of the micro-scale world with only experimental data. Bioinformatics-based analyzes such as molecular docking can contribute to the study of interactions, bindings and reactions between molecules and elucidate the mechanisms that occur in vivo. The possible mechanism of toxicity of AFM_1_ can be predicted by considering the interactions obtained by molecular docking and data from experimental steps together. In this study, AFM_1_-histone, AFM_1_-tubulin, AFM_1_-DNA interactions were analyzed by molecular docking in order to estimate the genotoxic and cytotoxic mechanism of AFM_1_.

## Material and methods

### Test materials and experiment protocol

**Figure 1:**
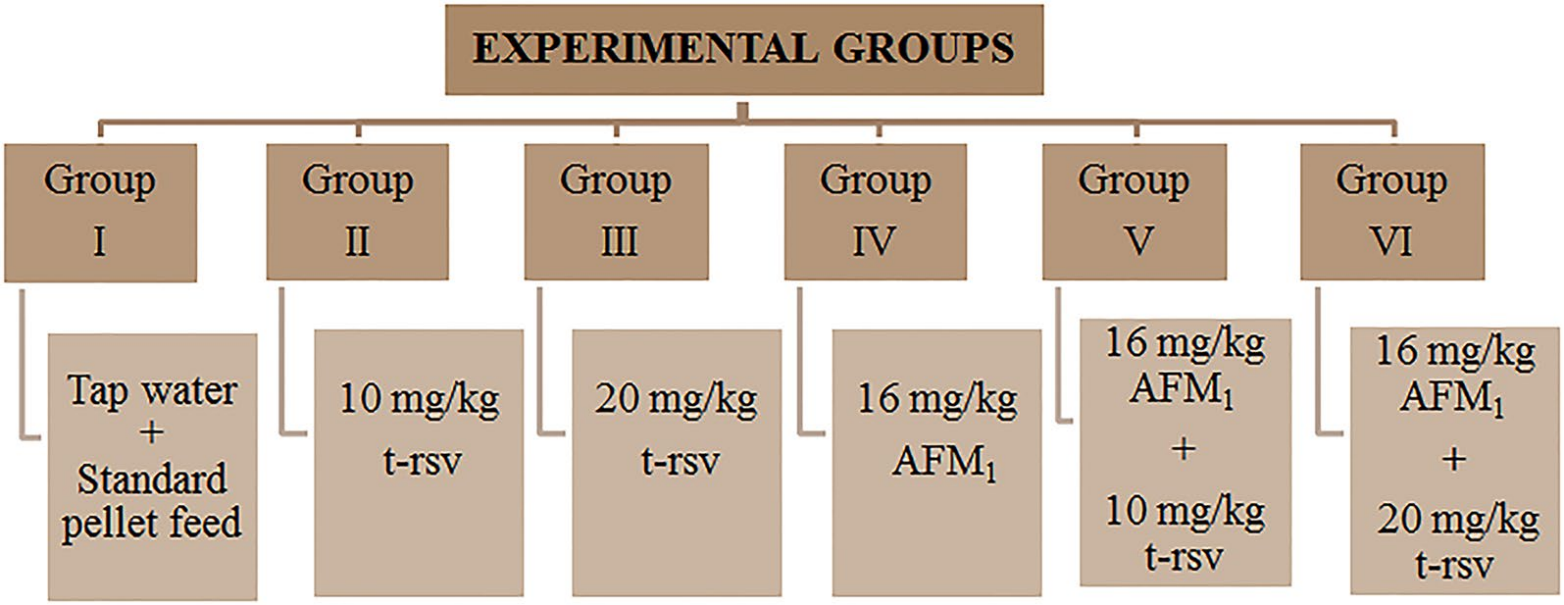
Experimental groups of the study.

In this study, 36 healthy male *Mus musculus* (12–14 weeks old, 25–30 g) were used as subjects and obtained from GRU-Experimental Animals Laboratory. Albino mice were maintained in stainless steel cages, at 22 ± 3 °C and 55 ± 5% relative humidity, 12 h light/12 h dark cycle. All experiments were performed in accordance with the guidelines of the Animal Experiments Local Ethics Committee of Giresun University and approved by the Animal Ethics Committee of Giresun University (protocol number: 2017/02). This study was carried out in compliance with the ARRIVE guidelines. Six groups were formed with six mice in each group. The groups and the application to which the group is exposed are given in Fig. 1.

In the literature, the LD_50_ value for AFM_1_ has been estimated in the range of 9–16 mg/kg, and the upper limit of 16 mg/kg was preferred in this study^13^. In the selection of t-rsv dose, the dose range in which resveratrol provides protection against chemicals in mice was preferred^14^. Mice were brought to the laboratory where the experimental stages would be conducted seven days ago to adapt to the environmental conditions. The water, feed, AFM_1_ and t-rsv solutions of each group were controlled daily. At the end of the 28-day treatment period, all mice were sacrificed. During the experiment, clinical symptoms of all animals such as activity, irritability, diarrhoea, wound formation and death were monitored daily.

### Changes in body weight, kidney and liver weights

After the mice were stunned under halothane anesthesia, their body weights were measured before the administration period and at the end of the 28-day administration period. In addition, at the end of the 28th day, the liver and kidney organs of the sacrificed mice were isolated and their weights were measured.

### Feed consumption

In order to interpret the changes in body weight and organ weights, the weekly feed consumption of the mice was measured. Albino mice can consume approximately 3–4 g of feed in a day^15^. In line with this information, 30 g of feed per day were put into the cages, with 5 g for each mouse in each group. Feed consumption was calculated by measuring the amount of feed remaining in the container at the end of the 7th day of each week during the 28-day application period.

### Analysis of serum parameters

Whole blood samples from mice stunned under halothane anesthesia were collected in vacuum tubes and centrifuged at 1200*g* at + 4 °C for 10 min. In serum obtained from blood, aspartate aminotransferase (AST, Teco Diagnostics, CAT. NO: A559–150), alanine aminotransferase (ALT, Teco Diagnostics, CAT. NO: A524–150), blood urea nitrogen (BUN, Teco Diagnostics, CAT. NO: B549–150) and creatinine (Teco Diagnostics) levels were measured on a Medispec 99M autoanalyzer using a commercial kit^16^.

### Changes in antioxidant/oxidant balance

To determine the effects of AFM_1_ and t-rsv on antioxidant/oxidant balance, malondialdehyde and glutathione levels were measured in the kidney and liver. For this purpose, liver and kidney organs were isolated from each mouse sacrificed under halothane anesthesia and washed with a sterile solution. Liver and kidney organs were homogenized with cold 0.15 M KCl solution. Homogenates were centrifuged at 5000*g* and 4 °C for 1 h and MDA and GSH analyzes were performed in the supernatant^17^.

### Cytotoxic and genotoxic effects

The genotoxic effects of AFM_1_ and the genotoxicity reducing effects of t-rsv were determined by MN and CAs analyses. MN test was performed in erythrocyte, buccal mucosa epithelium and leukocyte cells. CAs analyzes were investigated in bone marrow cells. The cytotoxic effects of AFM_1_ and the cytotoxicity reducing effects of t-rsv were determined by MI in bone marrow cells.

### MN test

For the MN test in the buccal mucosa epithelial cells, the mouths of the mice stunned with halothane anesthesia were rinsed with distilled water, and epithelial cells were taken from the left and right buccal mucosa and spread on the slide. Preparations fixed in methanol: acetic acid solution were stained with Fast Green and Feulgen then examined with a research microscope. For the MN test in erythrocytes, blood samples (5 µL) from the tail veins of mice stunned with halothane anesthesia were mixed with EDTA (3%) solution and spread on sterile slides. The preparations were fixed in ethanol (70%) for 2 min. were left to dry at 21 °C for 24 h. Slides were stained with Giemsa (5%) for 15 min. and analyzed under a microscope. For the leukocyte MN test, blood samples were obtained from each mouse and centrifuged at 5000 rpm for 10 min. 5 mL of 0.075 M KCl solution was transferred to the pellet. After incubation for 20 min., the solution was centrifuged at 5000 rpm for 10 min. and a washing solution consisting of 3:1 methanol/acetic acid was added and the mixture incubated at − 20 °C for 30 min. then leukocyte cells were spread and examined under a microscope after staining with Giemsa^4^.

### CA and MI analysis

CAs and MI analysis were determined in the bone marrow. For this aim, mice treated with 0.025% colchicine were sacrificed 2 h later under halothane anesthesia. Bone marrow obtained from the femurs of mice was aspirated, washed with the physiological solution and 0.075 M KCl was transferred. After fixation with Carnoy's solution, samples stained with Giemsa (5%) were examined under a microscope^18^. MI rates and CAs frequencies were determined in the prepared slides and 1.000 cells were analyzed for each group. In prepared slides, MI was determined as the percentage of dividing cells among 1000 nucleated cells for each group. Recovery effects (RE) of t-rsv against AFM_1_ induced genotoxicity were calculated by using Eq. (1). In determining RE, data belonging to Group VI, where t-rsv provided the highest healing effect, Group IV, in which AFM_1_ was treated alone, and the control group were used.

1 $$ {\text{RE }}\left( \% \right) \, = \, \left[ {\left( {{\text{D}}_{{1}} - {\text{D}}_{{2}} } \right) \, /\left( {{\text{D}}_{{3}} - {\text{D}}_{{2}} } \right)} \right] \, \times { 1}00 $$

D_1_: data of AFM1 + t-rsv treated group, D_2_: Data of AFM_1_ treated group, D_3_: data of control.

### Comet assay (single-cell gel electrophoresis)

The protocol of Tice et al.^19^ was performed for alkaline single cell gel electrophoresis with slight modifications. Slides were dipped in 1% normal melting point agarose for coating and allowed to dry at 37 °C. 10 µL of peripheral blood were added to 120 µL of 0.5% low-melting-point agarose at 37 °C, layered onto a coated slide, covered with a coverslip and left at 4 °C for 5 min. to solidify the agarose. The coverslip was removed and the slides were immersed into a lysis solution (2.5 M NaCl, 100 mm Na_2_EDTA, 10 mM Tris–HCl buffer, pH 10, 1% Triton X-100) for approximately 1 h. After lysis, the slides were transferred to a horizontal gel electrophoresis tank with a fresh and cooled alkaline buffer. After a 20 min. DNA unwinding period, electrophoresed at 0.86 V/cm (20 V, 300 mA) for 20 min. Slides were stained using ethidium bromide staining solution after carefully flushing three times with Tris-buffer (0.4 M Tris, pH 7.5) for 5 min. The preparations were washed with cold water to remove excess stain and covered with a coverslip. To prevent DNA damage, all steps were performed in low light and analyzed by fluorescence microscopy. Comets were analyzed with Comet Assay software version 1.2.3b^20^ with the parameters of tail DNA length. A total of 600 cells were analyzed for each group, 100 in each animal for DNA damage. The extent of DNA damage was scored from 0 to 4 depending upon the level of DNA damage. The cells were classified into five categories based on tail DNA length ranging from zero to four according to Collins^21^. The total DNA damage per group, expressed as arbitrary units, was calculated using Eq. (2).

2 $$Arbitrary \;unit= {\sum }_{i=0}^{4}Ni x i$$

(*i*: degree of damage (0, 1, 2, 3, 4), *Ni:* the number of cells in *i* degree).

### Molecular docking

Molecular docking studies were carried out to elucidate the mechanism of the cytotoxic and genotoxic effects of AFM_1_. For this purpose, potential interactions of AFM_1_ with DNA molecules, histone and tubulin proteins were investigated. The cyro-em 3D structure of tubulin (alpha-1B chain and tubulin beta chain) (6RZB)^22^, the crystal 3D structure of histone proteins (histone H3.1, histone H4, histone H2A and histone H2B type 1-A) (3X1T)^23^ and the 3D structures of B-DNA dodecamer (PDB ID: 1bna)^24^, B-DNA dodecamer d (PDB ID: 195d)^25^ and DNA (PDB ID: 1cp8)^26^ molecules were obtained from the protein data bank. The 3D structure of aflatoxin M_1_ (PubChem CID: 15558498) was retrieved from the PubChem. It was prepared for molecular docking by determining the active sites of proteins, removing water molecules and ligands, and adding polar hydrogen atoms. Energy minimization of proteins was done with Gromos 43B1 using Swiss-PdbViewer^27^ (v.4.1.0) software whereas energy minimization of the 3D structure of AFM_1_ was accomplished with the uff-force field employing Open Babel v.2.4.0 software^28^. Ligands in tubulin and histone proteins obtained from the protein data bank were saved as separate PDB files to validate the molecular docking procedure. Molecular docking validation was carried out by redocking these ligands to the active site of proteins with the AutoDock 4.2.6 software using the same docking protocol containing the grid parameters. Full binding of the inhibitor to the active site and less divergence than the complex obtained from the protein data bank confirms the protocol. In addition, the binding energy, overlay methods and chemical similarity of AFM1 to existing ligands were also considered. The receptor molecules were allocated Kollman charges, whereas AFM_1_ was assigned Gasteiger charges. The molecular docking process was carried out with the grid box containing the active sites of proteins and the entire structure of DNA molecules. Then docking was performed using Autodock 4.2.6 software^29^ based on Lamarckian genetic algorithm. The docking analysis and 3D visualizations were performed with Biovia Discovery Studio 2020 Client.

### Confirmation of the DNA-AFM_1_ interactions by spectral measurements

Spectral measurements were performed to confirm the DNA-AFM_1_ interactions. For this purpose, DNA was first obtained from the blood of mice. The cetyltrimethyl ammonium bromide (CTAB) method recommended by Miladinov^30^ was used for DNA extraction from blood. Whole blood samples were taken from the tail veins of the mice with the help of a fine-tipped syringe. The samples were transferred to 2 mL tubes containing EDTA. 500 µL of solution I (8% CTAB, 1.5 M NaCl, 100 mM TRIS pH 8.5, 50 mM EDTA pH 8) pre-warmed at 68 °C was mixed with 250 µL of blood sample and incubated at 68 °C for 30 min. Then, 750 µL of chloroform was added and mixed by inverting several times then centrifuged for 5 min. at 13,000 rpm at 21 °C. The volumes were determined by transferring the DNA containing the upper phase and the proteins containing the middle phase to new tubes without deterioration. The aqueous phases were mixed with solution II (5% CTAB, 0.1 M NaCl) and dH_2_O and centrifuged at 13,000 rpm for 5 min at 21 °C. Supernatants were discarded and pellets were re-suspended in 250 µL of 1.2 M NaCl. 750 µL of ice-cold absolute ethanol was added to precipitate DNA and the samples were centrifuged for 5 min at 13,000 rpm at + 4 °C. The pellets were washed twice with 750 µL of 70% ethanol and centrifuged under the above conditions to remove residual salt. The tubes were incubated at 55 °C for approximately 2 h with the caps open to allow the excess ethanol to evaporate. In the final step of isolation, DNA pellets were suspended in 50 µL of TE buffer (10 mM Tris–HCl, 1 mM EDTA, pH 7.6) and stored at − 20 °C until analysis.

The DNA solution was prepared by gentle shaking in 0.01 M sodium nitrate solution. DNA-AFM_1_ interaction was evaluated by investigating the change in absorbance of mixtures containing DNA and different concentrations of AFM_1_ (1:1, 1:2, 1:4). The UV absorption spectrum of DNA-AFM_1_ complex in the range of 220–300 nm was obtained^31^. UV absorption spectra were recorded on the Mapada UV-6100PCS double beam spectrophotometers.

### Statistical analysis

Statistical analysis of the data obtained from experimental stages was carried out using the SPSS for Windows V 22.0 (SPSS Inc, Chicago, IL, USA) package program. One-way ANOVA and Duncan tests were used to evaluate the statistical differences between the experimental groups, respectively. Obtained data were shown as mean ± SD and were considered statistically significant when p values were < 0.05. Pearson correlation analysis (two-sided) was performed in RStudio and correlation plots were performed with the corrplot package^32^. Principal component analysis (PCA) was performed for physiological, biochemical and genetic parameters, which are different biomarkers of toxicity for each dose tested. The FactoMineR^33^ and factoextra^34^ packages in RStudio were used to perform principal component analysis (PCA)^35^.

## Results and discussion

### Alterations in body and organ weights

The effects of AFM_1_ and t-rsv on the body and organ weights in albino mice are given in Table 1. Body weights of Groups I, II and III increased significantly at the end of the application period. There was no statistically significant difference between these groups in terms of body weight gain (p > 0.05) and an increase in body weight in the range of 12.4–11.62 g was recorded for these three groups. Body weight, liver and kidney weights decreased 17.5%, 43.1% and 51.3%, respectively, in the AFM_1_ applied group compared to the control group. These reductions are directly related to the feed intake of the mice. Feed consumption of each group was followed for 28 days. There was no statistically significant change in feed consumption for 28 days in Groups I, II and III. In the AFM_1_ treated group, feed consumption was found to be similar on the 7th and 14th days, but decreased significantly on the 21st and 28th days compared to control. The adverse effects of AFM_1_ application on body and organ weights are directly related to the reduction in feed intake as well as indirectly related to impaired protein/lipid metabolism, anorexia, inhibition of lipogenesis and protein synthesis. Lipogenesis-lipolysis balance has an important role in increasing body weight and especially lipogenesis induces weight gain^36^. Lipid metabolism abnormalities, which may occur as a result of toxic effects of aflatoxins on the liver and other tissues, significantly affect the weight gain and organ weights of organisms. Although there is no study on the effect of AFM_1_ on weight gain and feed intake in albino mice, there are important data with other aflatoxin types. Arvind and Churchil^37^ reported weight gain of chickens fed with AFB_1_ was reduced 33.94% compared to control. Dimitri et al.^38^ determined that AFB_1_ and AFG_2_ treatments caused a body weight loss of approximately 539 g in rabbits compared to the control group, and this loss was associated with disruptions in protein and DNA synthesis. Hussain et al.^39^ determined that AFB_1_ administration caused depression, decrease in feed intake, body weight and defecation in rats. Contrary to our findings, Casado et al.^40^ stated that AFB_1_ administration did not cause any change in feed consumption and weight gain in mice.

**Table 1:** The effects of AFM_1_ and t-rsv on weight parameters and feed consumption.

Parameters		Group I	Group II	Group III	Group IV	Group V	Group VI
Body weight	IBW	38.54 ± 1.92	37.95 ± 1.86	38.26 ± 1.88	37.88 ± 1.84	38.30 ± 1.90	37.90 ± 1.83
	FBW	50.16 ± 2.13	49.91 ± 2.10	50.66 ± 2.18	41.38 ± 1.98	43.92 ± 2.11	44.90 ± 2.16
	TWG	11.62^a^	11.96^a^	12.40^a^	3.50^d^	5.62^c^	7.00^b^
O.W	Liver	2.64 ± 0.38^a^	2.62 ± 0.34^a^	2.65 ± 0.42^a^	1.50 ± 0.24^d^	1.78 ± 0.27^c^	2.16 ± 0.34^b^
	Kidney	1.52 ± 0.28^a^	1.55 ± 0.30^a^	1.53 ± 0.26^a^	0.74 ± 0.15^d^	0.92 ± 0.18^c^	1.24 ± 0.22^b^
F.C.	7th day	149.5	151.9	157.6	148.6	146.7	147.8
	14th day	150.3	152.7	153.2	147.1	143.9	144.7
	21st day	153.2	155.7	155.6	131.1	133.8	148.1
	28th day	152.9	153.2	151.9	119.8	140.6	149.6

Group I: Control, Group II: 10 mg/kg b.w. t-rsv, Group III: 20 mg/kg b.w. t-rsv, Group IV: 16 mg/kg b.w. AFM_1_, Group V: 10 mg/kg b.w. t-rsv + 16 mg/kg b.w. AFM_1_, Group VI: 20 mg/kg b.w. t-rsv + 16 mg/kg b.w. AFM_1._ Values are shown as mean ± SD (n = 6). The averages shown with different letters^(a–d)^ on the same line are statistically significant (p < 0.05). IBW: Initial body weight (g), FBW: Final body weight (g), TWG: Total weight gain (g), O.W: organ weights, F.C: feed consumption.

Treatment of t-rsv with 16 mg/kg b.w. AFM_1_ resulted in an improvement in body weight and organ weights compared to Group IV. While 3.5 g weight gain was recorded at the end of the 28th day in AFM_1_ treated group, an increase of 5.62 g and 7 g in body weights were detected in Groups V and VI in which t-rsv and AFM_1_ were administered together. Although these increases lagged behind the control group, a significant improvement was achieved compared to the only-AFM_1_ treated group. The positive effects of t-rsv on body weight gain were also observed in organ weights. In Group VI treated with t-rsv and AFM_1_, liver and kidney weights increased by 30.5% and 40.3%, respectively, compared to the AFM_1_ treated group. Similar increases were observed in feed intake, and feed consumption increased significantly in Group V and Group VI compared to the AFM_1_-treated group. T-rsv administration provided significant protection against a decrease in body and organ weights, and this protection was statistically significant especially at 20 mg/kg dose compared to the permethrin-only group (p < 0.05). This curative property of t-rsv on weight gain and feed consumption can be explained by the suppression of the toxic effects exhibited by AFM_1_. Resveratrol has an important effect on lipid metabolism in organisms. Resveratrol prevents oxidative stress-induced LDL oxidation and lipid peroxidation. It also plays an important role in lipid metabolism by decreasing low-density lipoprotein and total cholesterol levels and increasing plasma high-density lipoprotein levels. The regulatory role of resveratrol in lipid metabolism and its activity to repair damage in liver and kidney tissues are the most important factors of recovery in body and organ weights^4^. In the literature, it has been reported that resveratrol application improves the changes in body and liver weights observed in organisms under the influence of various exogenous factors^41,42^.

### Antioxidant and oxidant dynamics

**Figure 2:**
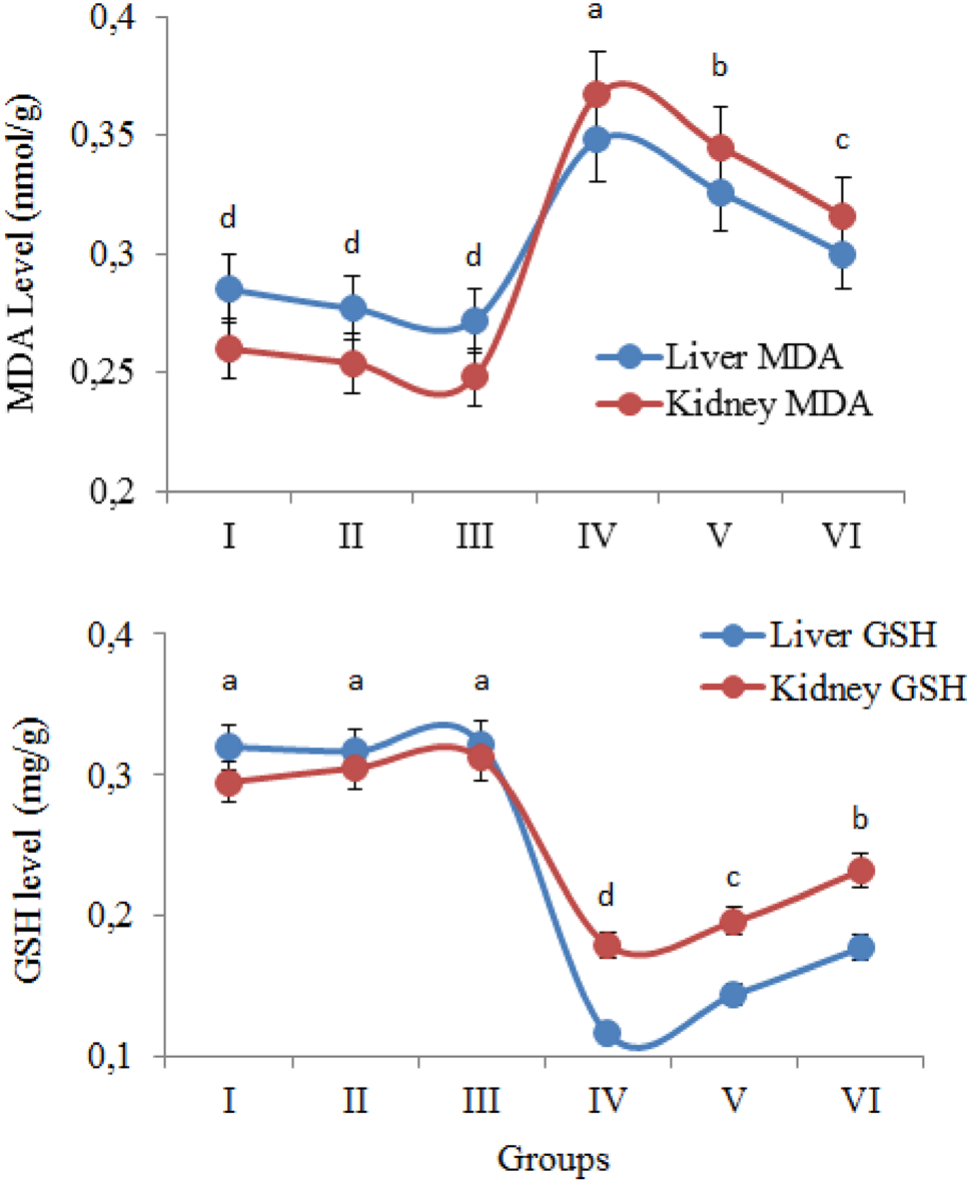
The effect of AFM_1_ and t-rsv on MDA and GSH levels. Group I: Control, Group II: 10 mg/kg b.w. t-rsv, Group III: 20 mg/kg b.w. t-rsv, Group IV: 16 mg/kg b.w. AFM_1_, Group V: 10 mg/kg b.w. t-rsv + 16 mg/kg b.w. AFM_1_, Group VI: 20 mg/kg b.w. t-rsv + 16 mg/kg b.w. AFM_1._ Different letters^(a–d)^ indicated averages p < 0.05 are significant.

In order to investigate the effects of AFM_1_ and t-rsv applications on antioxidant/oxidant balance in liver and kidney tissues, GSH and MDA levels were measured and the results are given in Fig. 2. MDA and GSH levels were found to be similar in liver and kidney tissues in the control group and only t-rsv treated groups. Abnormal increases in MDA levels were detected in the group treated with 16 mg/kg AFM_1_. MDA levels of the liver and kidney increased 1.22 and 1.41 times, respectively, in the AFM_1_ applied group compared to the control group. MDA normally occurs at low levels in cells and is involved in various biochemical reactions. MDA, which is formed as a result of lipid peroxidation, is a mutagenic agent and is used as an indicator in the measurement of oxidative damage. Increased MDA level in a cell indicates the presence of oxidative stress. The most important cellular structures affected by oxidative stress are cell membrane lipids, proteins and DNA. The increased MDA level in the liver and kidney of the AFM_1_ applied groups indicates the oxidation of lipids in the cell membrane, and the high level of MDA formed as a result of oxidation also induces DNA damage^43,44^. Similarly, Shen et al.^45^ reported that AFB_1_ administration induces lipid peroxidation and causes cell damage in rat liver cells. The increase in the level of oxidant molecules in the cells causes a decrease in the levels of endogenous antioxidants and the deterioration of the antioxidant/oxidant balance. The significant decrease in GSH levels in the liver and kidney tissues in AFM_1_ treated group confirms this hypothesis. 16 mg/kg AFM_1_ application decreased the GSH levels in the liver and kidney by 63.7% and 39.3%, respectively, compared to the control group. GSH, which has a tripeptide structure, is a non-enzymatic antioxidant and provides neutralization of free radicals in cells. In cells, glutathione can be found in two different forms: reduced (GSH) and oxidized (GSSG). The balance and ratio between reduced glutathione and oxidized glutathione in cells are used to evaluate the cellular oxidative damage^46,47^. In healthy cells and tissues, more than 90% of the total glutathione is in reduced form. The decrease in the reduced glutathione level indicates the presence of oxidative stress in the cell and the deterioration of the antioxidant/oxidant balance. As a result, increased MDA and decreased GSH levels after AFM_1_ treatment in the liver and kidney confirm that AFM_1_ is an important inducer of oxidative stress and disrupts the antioxidant/oxidant balance.

Within the scope of antioxidant/oxidant dynamic analysis, it was determined that t-rsv administration caused an improvement in antioxidant/oxidant balance, which was impaired by AFM_1_. AFM_1_ + 20 mg/kg t-rsv administration provided 34.4% and 22.8% improvement in liver and kidney GSH levels compared to the AFM_1_-treated group. The dose-dependent increase in GSH level and 16% decrease in MDA level are indications that t-rsv provides an improvement in the antioxidant/oxidant balance in the liver and kidney. Resveratrol reduces oxidative stress in the cell by different mechanisms and protects cellular structures. Some of these mechanisms are direct suppression of free radicals, induction of the activity of antioxidant enzymes such as glutathione peroxidase, superoxide dismutase and catalase. Resveratrol also reduces lipid peroxidation by neutralizing free radicals and preserves cell membrane integrity^48^. All these effects of resveratrol reduced the deterioration in antioxidant/oxidant dynamics induced by AFM_1_. While there is no result in the literature regarding the effect of t-rsv against oxidative damage induced by AFM_1_, it has been reported to be protective against oxidative damage caused by various chemicals. Şener et al.^49^ reported that administration of 30 mg/kg resveratrol in rats regulated the impaired antioxidant balance and caused an improvement in the decreased GSH level.

### Serum parameters

**Figure 3:**
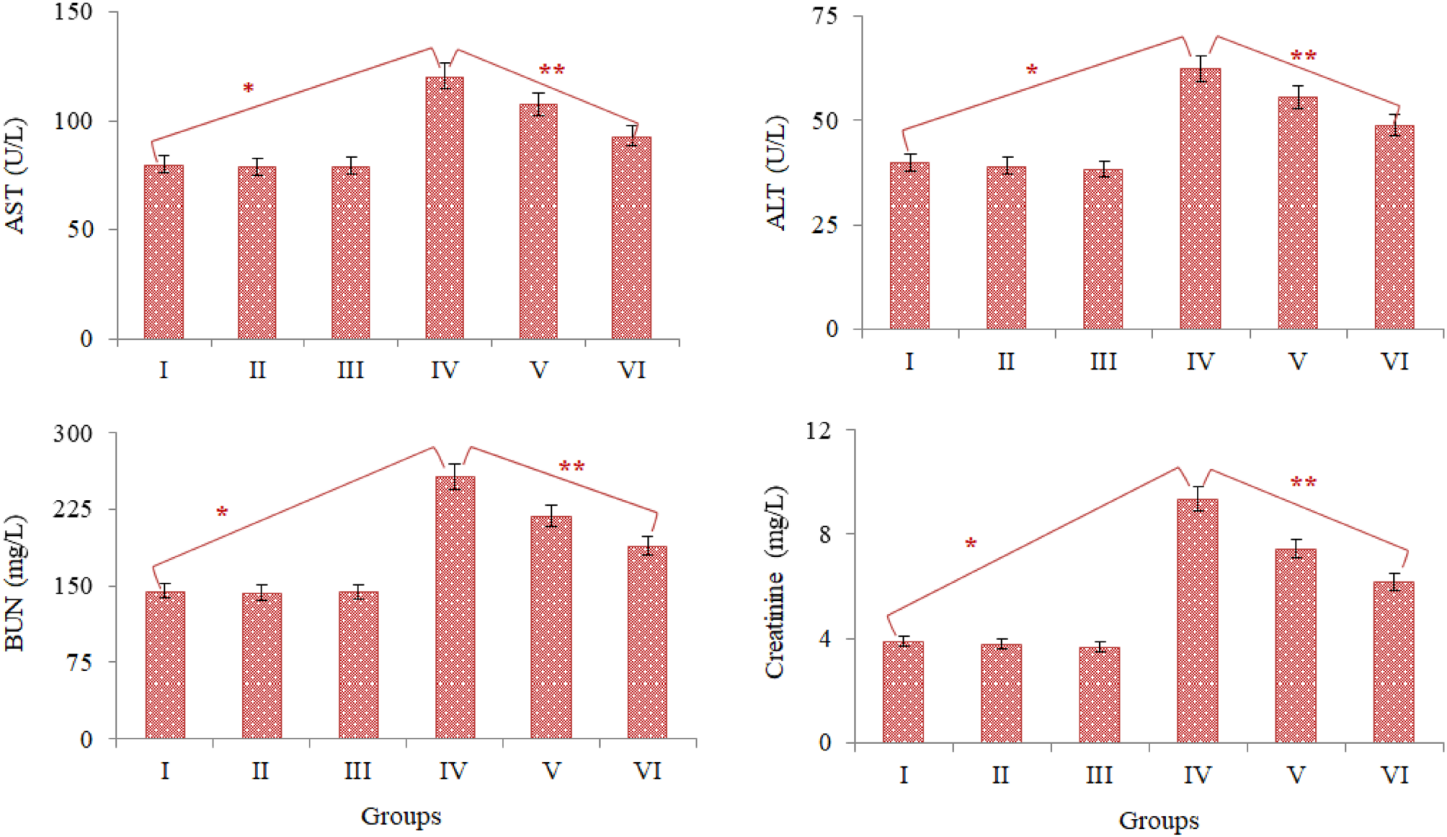
The effect of AFM_1_ and t-rsv on serum parameters. Group I: Control, Group II: 10 mg/kg b.w. t-rsv, Group III: 20 mg/kg b.w. t-rsv, Group IV: 16 mg/kg b.w. AFM_1_, Group V: 10 mg/kg b.w. t-rsv + 16 mg/kg b.w. AFM_1_, Group VI: 20 mg/kg b.w. t-rsv + 16 mg/kg b.w. AFM_1._ Values are shown as mean ± SD (n = 6).*indicates the statistical difference between Groups I and IV, **indicates statistical difference between Groups IV and VI (p < 0.05).

The effects of AFM_1_ and t-rsv application on serum parameters are given in Fig. 3. ALT and AST enzyme activities, which are indicators of liver cell damage, creatinine and BUN, which are accepted as indicators of kidney cell damage, were investigated. No difference was observed in terms of serum parameters examined in Groups I, II and III. This result shows that t-rsv administered alone did not cause a statistically significant difference in serum parameters. There were significant increases in all tested parameters in Group IV, which was administered 16 mg/kg AFM_1_. After AFM_1_ administration, AST, ALT, BUN and creatinine levels increased by 33.6%, 35.8%, 43.6% and 58.4%, respectively, compared to the control group. These results show that AFM_1_ administration causes damage to liver and kidney tissues, especially the damage to kidney tissue occurs at a higher rate. AST is found in many tissues such as the liver, lung, kidney, brain, heart, pancreas and skeletal muscle. AST is an intracellular enzyme and its serum levels are quite low. After damage occurs in the tissues where AST is present, it passes from the damaged tissue cells to the blood, and the serum level also increases. Although a high AST level in the serum is an indicator of tissue damage, it is not sufficient for the detection of liver damage alone. ALT is concentrated in the liver and is therefore considered a direct indicator of liver damage^50^. It is known that aflatoxin species cause hepatocellular necrosis, inhibition of polymerase activity, biochemical and pathological changes in liver cells^51^. As a result of these abnormalities caused by aflatoxins, cell damage occurs, enzymes leak into the blood and their levels increase. The increase in both AST and ALT levels together in this study is an indication that AFM_1_ exposure induces liver damage. Although there is no study in the literature investigating the effects of AFM_1_ on the liver or liver markers, other types of aflatoxins are reported to have similar effects. Han et al.^52^ reported that administration of 20–40 μg/kg AFB_1_ caused liver damage and significant increases in serum ALT and AST levels. In this study, similar to increases in AST and ALT, increases in BUN and creatinine levels were also observed after 20 µg/kg AFM_1_. Creatinine, which is formed as a breakdown product of creatine phosphate in muscle tissue, is excreted from the body by the kidneys. The amount of nitrogen in urea formed as a result of protein catabolism is expressed as BUN. Urea formed in the liver is removed from the body through the kidneys in the urine. It is known that creatinine and BUN levels increase in conditions such as kidney diseases, obstruction of the urinary tract and kidney stones^53^. Aflatoxins cause toxic effects in kidney tissue such as damage to glomerular capillaries, occlusion of cortical blood vessels, inflammation, coagulation necrosis, focal bleeding and occlusion areas^54^. These damages cause an increase in BUN and creatinine in the blood. The significant increases in BUN and creatinine levels observed in this study indicate that 16 mg/kg b.w. AFM_1_ exposure causes renal damage. Eraslan et al.^55^ reported that administration of 500 μg/kg aflatoxin in albino rats induced kidney damage, resulting in significant increases in BUN, creatinine and uric acid levels.

In this study, it was also determined that abnormal increases in serum parameters regressed with AFM_1_ + t-rsv administration. This regression indicates the protective property of t-rsv and this protection increases depending on the dose. AST, ALT, BUN and creatinine levels in Group V administered with 10 mg/kg t-rsv + AFM_1_ decreased by 11.2%, 10.7%, 14.9% and 20.5%, respectively, compared to AFM_1_-treated group. Same improvements in 20 mg/kg t-rsv + AFM_1_ treated group (Group VI) were 22.5%, 21.6%, 26.2% and 34.2%, respectively. As the t-rsv dose increased, the improvements in serum parameters also increased and the differences between Group V and Group VI were statistically significant in terms of each serum parameter (p < 0.05). The improvements in serum parameter levels after t-rsv administration proves the protective effect of t-rsv on liver and kidney tissue. The results of the antioxidant and oxidant dynamics analysis of this study revealed that AFM_1_ causes oxidative stress. Oxidative stress-induced by AFM_1_ causes significant damage to liver and kidney tissues. Resveratrol provides protection by preventing the oxidation of macromolecules in the liver and kidney^56^. Although there is no study in the literature on the healing effects of t-rsv against AFM_1_-induced damage, it is reported that resveratrol decreases damage in the liver and kidney induced by chemical agents. Akosman et al.^57^ reported that 40 mg/kg resveratrol administration provided significant protection against liver and kidney injuries.

### Analysis of genotoxic effects

**Figure 4:**
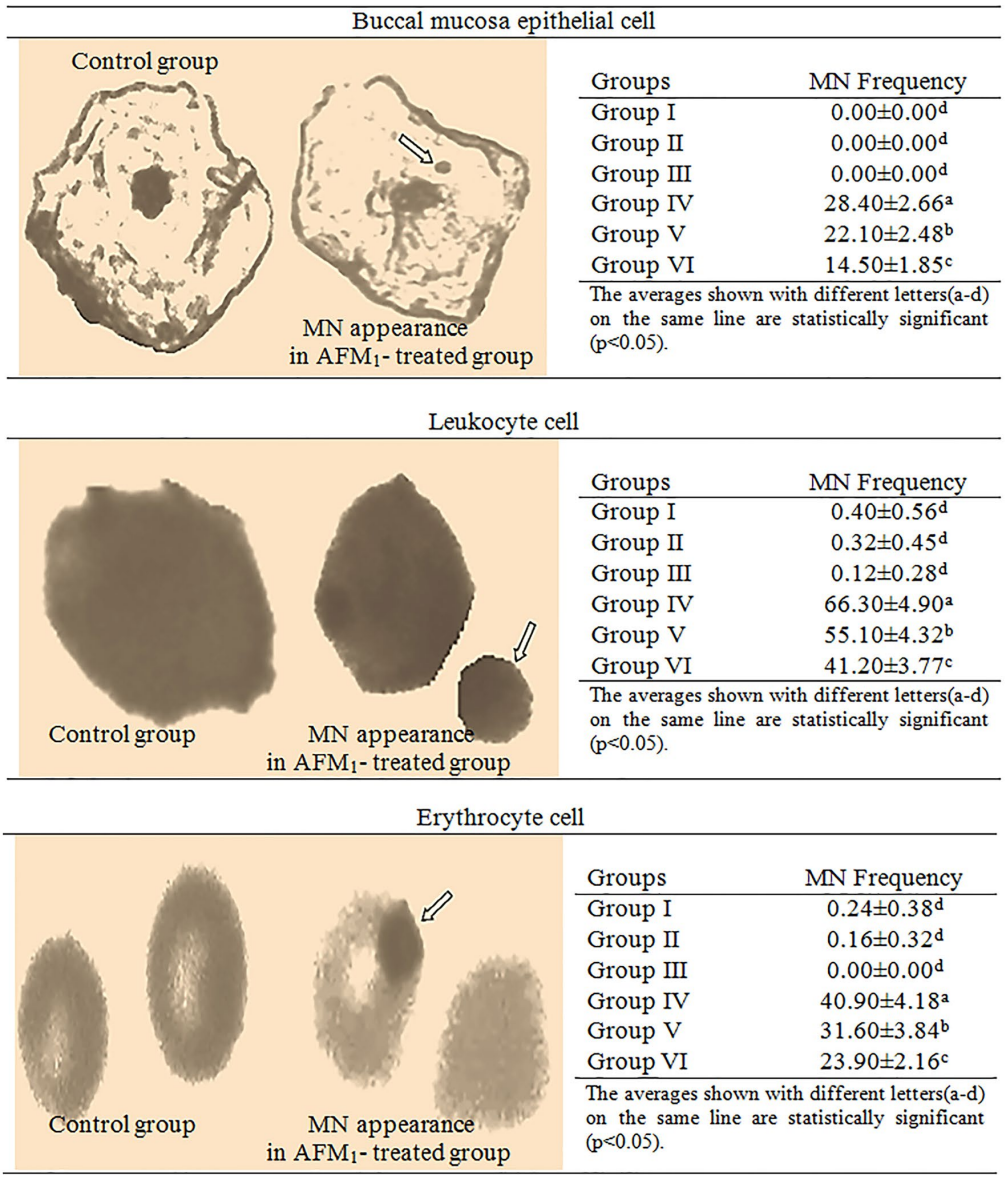
MN formation induced by AFM_1_. 1000 cells in each group were analyzed for MN frequency.

The genotoxic effects of AFM_1_ and the protective role of t-rsv were investigated by MN and CAs analyses. MN formations were investigated in leukocyte, erythrocyte and buccal mucosa cells, CAs were investigated in bone marrow cells. The effects of AFM_1_ and t-rsv applications on MN frequency are given in Fig. 4. While no MN formation was observed in buccal epithelial cells, statistically insignificant MN formation was detected in leukocyte cells in control and only-t-rsv applied groups (p > 0.05). Negligible levels of MN were observed in the erythrocyte cells of the control group and 10 mg/kg t-rsv treated group (p > 0.05). This similarity between t-rsv-treated groups and the control group indicates that t-rsv does not have an inducing effect on MN formation. Significant levels of MN formation were detected in the erythrocyte, buccal epithelium and leukocyte cells of the AFM_1_-applied group. Among the cells, the highest MN formation was observed in leukocyte cells and the lowest in buccal epithelial cells. MN formation in a cell indicates the genotoxic effects and the presence of an agent that induces this effect. MNs can arise from single-stranded and double-stranded DNA breaks or lagging chromosomes. A nuclear membrane forms around these formations and MNs appear, similar in structure to the main nucleus, stained in the same color but smaller in size^58^. The formation of MNs is due to aneugenic or clastogenic effects, and the size of the MN also provides information about the type of these effects. Aneugenic agents cause centromere division errors, defects on spindle apparatus and lagging chromosomes, resulting in larger MN formations. Clastogenic agents cause DNA chain breaks, acentric fragments or chromosome breaks, resulting in the formation of MN in smaller sizes^59^. While the presence of MN indicates a genotoxic effect in the cell, the size of MN provides information about the mechanism of this effect. AFM_1_ exposure induced large MN in leukocyte and erythrocyte cells and smaller MN in buccal mucosa cells. This result shows that AFM_1_ exhibits both aneugenic and clastogenic effects by causing spindle apparatus defects, centromere division errors, acentric fragments or chromosome breaks. Similarly, Corcuera et al.^60^ reported that AFB_1_ administration induced MN formation in bone marrow cells in rats.

The CAs types observed in Group IV-treated with AFM_1_ confirm that AFM_1_ has both aneugenic and clastogenic effects. The effects of AFM_1_ and t-rsv on CAs frequency in bone marrow cells are given in Table 2. No statistically significant CAs formation was found in the control group and only t-rsv administered groups. Negligible fragment formation was observed only in the 10 mg/kg t-rsv treated group. AFM_1_ application caused significant CAs formations with high frequency. Chromosome breaks are the most common type of CAs and fragment, gap, ring, acentric and dicentric chromosome abnormalities are also other types of CAs induced by AFM_1_. Both MN formations and the observation of different types of CAs indicate the genotoxic effects of AFM_1_. The size of MNs induced by AFM_1_ provides information about the mechanism of toxicity; also the types of CAs give information about the genotoxicity mechanisms of AFM_1_. The high frequency of break, ring and fragment formation in bone marrow cells indicates the clastogenic effect of AFM_1_. As a result of the clastogenic effect, DNA chain breaks or chromosome breaks occur and these abnormalities cause the formation of other CA types. Chromosomes recombine at their breaking points and turn into ring chromosomes. Ring chromosomes that cannot be pulled to the poles in mitosis cause chromosome loss or excess in cells, resulting in high MN frequency^61^. Acentric and dicentric chromosomes are formed as a result of chromosome breaks. A dicentric chromosome is an abnormal chromosome with two centromeres and is formed by the fusion of segments originating from two chromosome breaks containing a centromere. As a result of the fusion of broken chromosome parts, dicentric chromosomes may occur, as well as acentric parts without a centromere^62^. When CA types induced by AFM_1_ are examined, it can be said that a high frequency of chromosome breaks is induced and other CA types are formed as a result of the re-arrangement of chromosomal breaks. While no study on the genotoxicity of AFM_1_ in albino mice has been reported in the literature, there are studies investigating the effects of other aflatoxin species. Fetaih et al.^63^ reported that AFB_1_ administration causes macro-DNA damages such as gaps, breaks, deletions, dicentric chromosomes, adherent chromosomes, hypopolyploidy, centromeric rearrangements in rats.

**Table 2:** The effect of AFM_1_ and t-rsv administration on CAs frequency in bone marrow cells.

CAs	Group I	Group II	Group III	Group IV	Group V	Group VI
Break	0.00 ± 0.00^d^	0.00 ± 0.00^d^	0.00 ± 0.00^d^	60.20 ± 4.38^a^	45.30 ± 4.12^b^	31.90 ± 3.18^c^
Ring	0.00 ± 0.00^d^	0.18 ± 0.00^d^	0.00 ± 0.00^d^	48.40 ± 4.15^a^	36.50 ± 3.70^b^	22.60 ± 2.84^c^
Fragment	0.00 ± 0.00^d^	0.00 ± 0.00^d^	0.00 ± 0.00^d^	31.80 ± 3.12^a^	24.60 ± 2.45^b^	17.80 ± 1.68^c^
Gap	0.00 ± 0.00^d^	0.00 ± 0.00^d^	0.00 ± 0.00^d^	20.80 ± 2.65^a^	12.40 ± 1.78^b^	7.50 ± 1.14^c^
Acentric	0.00 ± 0.00^d^	0.00 ± 0.00^d^	0.00 ± 0.00^d^	12.70 ± 0.94^a^	7.10 ± 0.58^b^	5.50 ± 0.46^c^
Dicentric	0.00 ± 0.00^d^	0.00 ± 0.00^d^	0.00 ± 0.00^d^	8.20 ± 0.84^a^	4.40 ± 0.62^b^	3.30 ± 0.38^c^

Values are shown as mean ± SD (n = 6). 600 cells were analyzed for chromosomal damage. The means indicated by different letters (a–d) on the same line are statistically significant (p < 0.05).

Another important result obtained from genotoxicity studies is that t-rsv treatment has a dose-dependent protective role in reducing the genotoxic effects. 10 mg/kg and 20 mg/kg t-rsv treatment with AFM_1_ resulted in a 22.6–63.9% reduction in CA types. The most significant reduction was observed in gap formation, and 20 mg/kg t-rsv application reduced gap formation by 63.9%. Considering that other CA types are generally thought to originate from chromosomal breaks, the decrease in break frequencies caused a decrease in other CA types as well. This genotoxicity-limiting effects of t-rsv can be associated with its antioxidant activity. Resveratrol is a molecule that neutralizes free radicals and potently induces intracellular antioxidants such as glutathione and catalase. Resveratrol, with its regulatory role on the cell cycle, suppresses the division of damaged cells and provides an opportunity for the cell to remain in the G_0_/G_1_ phase and repair the damage. In this way, it provides repair of chromosomal damages, prevents the division of damaged cells and reduces the frequency of CAs^64,65^. Although there is no study in the literature investigating the protective feature of t-rsv against AFM_1_ toxicity, its protective feature against genotoxicity has been demonstrated by many studies. Carsten et al.^66^ reported a significant reduction in the rate of radiation-induced chromosomal damage after resveratrol administration in mouse bone marrow cells.

### DNA fragmentation

**Figure 5:**
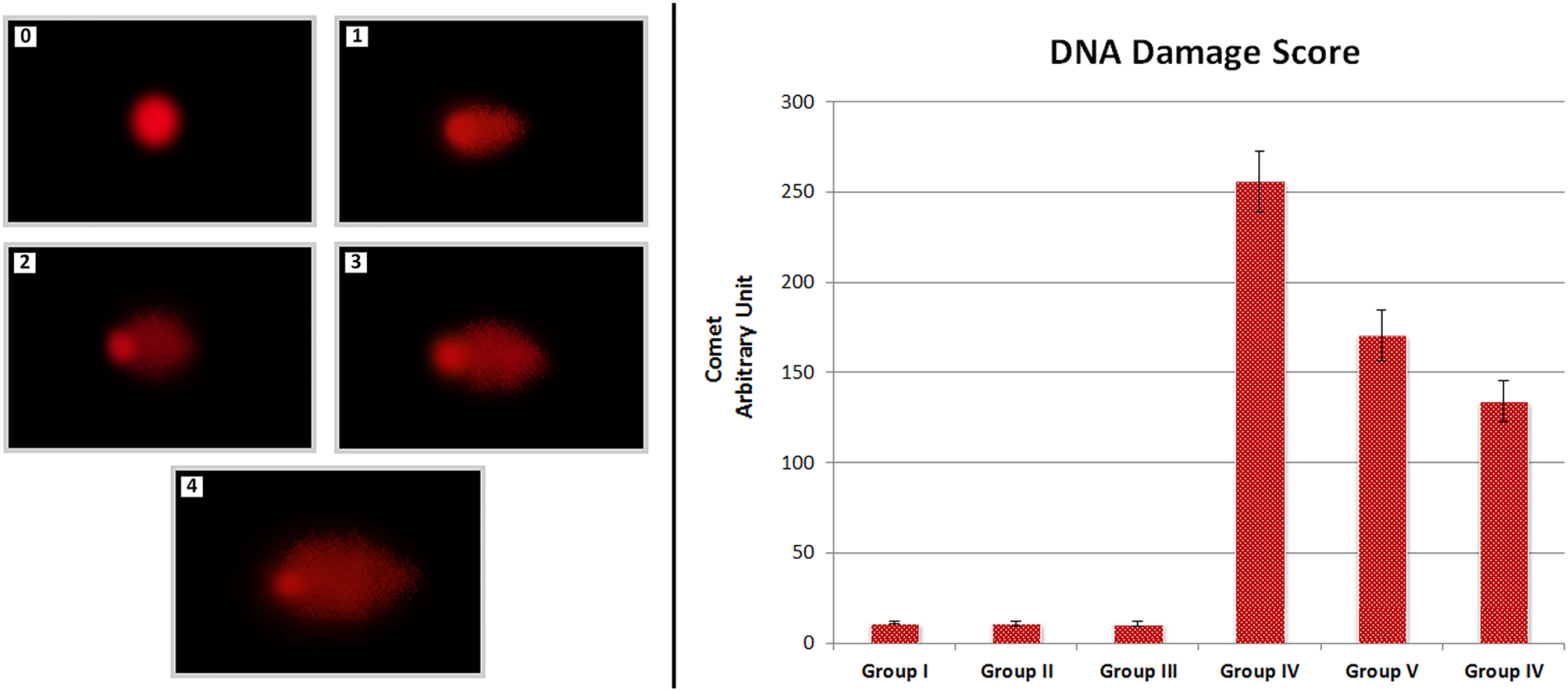
The effect of AFM_1_ and t-rsv application on leukocyte cell nuclei of swiss albino mice (0: no damage, 1: low damage, 2: moderate damage, 3: high damage, 4: extreme damage. Group I: control, Group II: 10 mg/kg bw t-rsv, Group III: 20 mg/kg bw t-rsv, Group IV: 16 mg/kg bw AFM_1_, Group V: 16 mg/kg bw AFM_1_ + 10 mg/kg bw t-rsv, Group VI: 16 mg/kg bw AFM_1_ + 20 mg/kg bw t-rsv).

The effects of AFM_1_ and t-rsv on DNA fragmentation were investigated by Comet assay. DNA fragmentation in the comet assay was evaluated on the basis of DNA damage score. Comet analysis data of AFM_1_ and t-rsv applied groups are given in Fig. 5. T-rsv application alone did not induce DNA damage, and no statistically significant difference (p > 0.05) was found between the control group (Group I) and the t-rsv alone treatment groups (Group II and III). AFM_1_ treatment was induced DNA damage in leukocyte cell nuclei of swiss albino mice, as evidenced by the results. While the average DNA damage score in Group I (control group) was 11.17 ± 0.79, the average DNA damage score in Group IV, which was treated with 16 mg/kg AFM_1_, was 255.67 ± 16.57. T-rsv treatment in addition to AFM_1_ had a dose-dependent protective effect. DNA damage score was 170.50 ± 14.26 in Group V administered with 16 mg/kg AFM_1_ + 10 mg/kg t-rsv and 133.83 ± 11.43 in Group VI administered 16 mg/kg AFM_1_ + 20 mg/kg t-rsv. The results demonstrated that AFM_1_ treatment-induced DNA fragmentation, but t-rsv treatment had a dose-dependent protective effect. There are statistically significant differences in DNA damage scores between Groups I-III and IV-VI (p < 0.05).

### Molecular docking and spectral measurements of AFM_1_-DNA interactions

**Figure 6:**
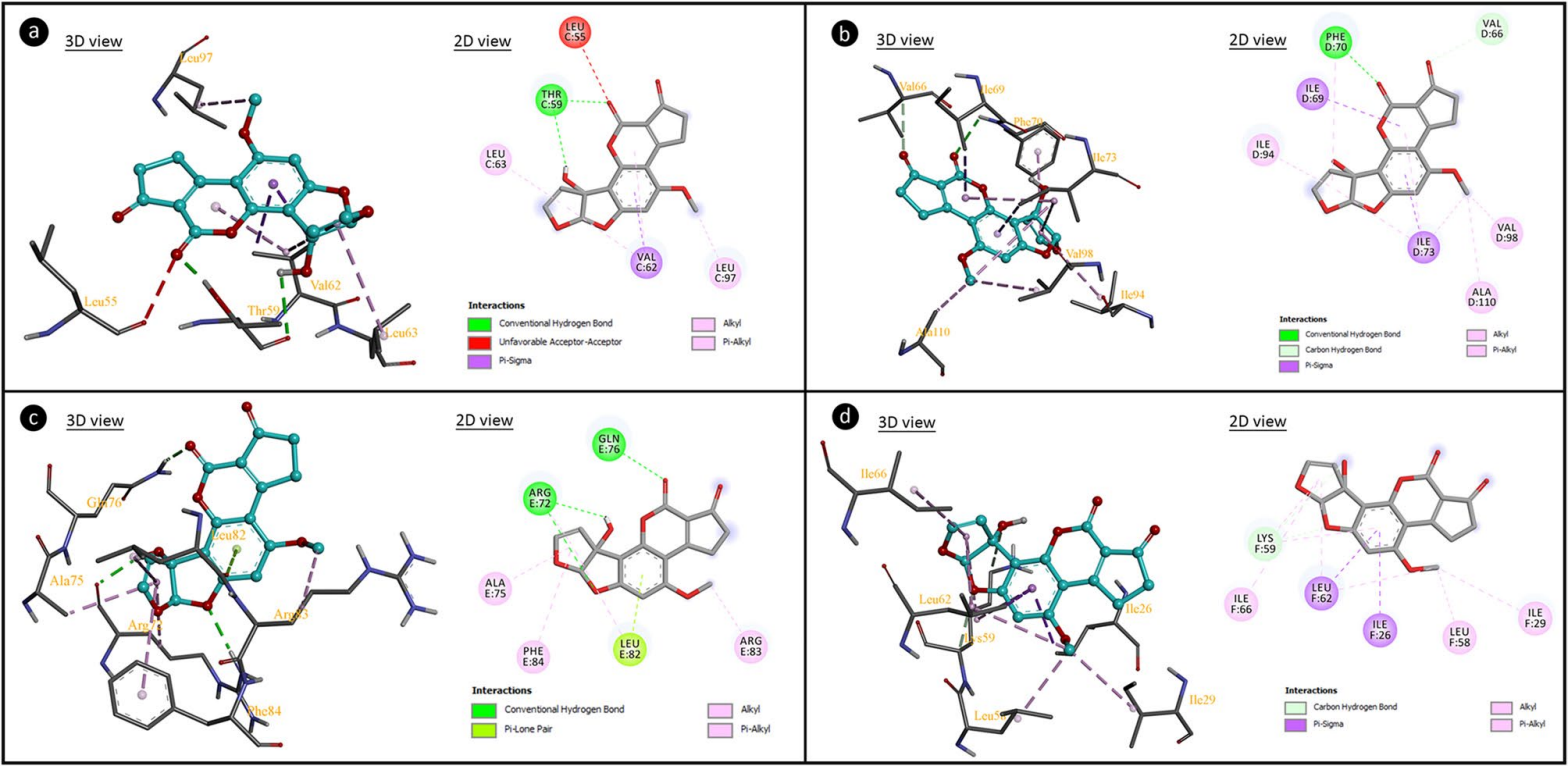
The molecular interactions of AFM_1_ with histone proteins (**a**: histone H3.1, **b**: histone H4, **c**: histone H2A, **d**: histone H2B type 1A).

As a result of CAs analysis, AFM_1_ application caused abnormalities in the chromosome structures. Molecular docking of AFM_1_ and histone proteins were performed to predict the possible mechanism of AFM_1_ genotoxicity. Histones are proteins that allow DNA to condense properly into chromosomes. Multiple hydrogen bonds, hydrophobic interactions and ionic bonds allow histones to bind to DNA. These bonds are usually formed between the amino acid backbones of histones and the sugar-phosphate backbone of DNA. Weakening of these bonds between histone proteins and DNA or deformations in the histone structure cause instability in the genome structure. Modifications in histone proteins affect different processes in the cell such as chromosome packaging, DNA damage and DNA repair^67^. Molecular docking analysis including inhibition coefficients and the binding energy of AFM_1_ and histone proteins are given in Fig. 6 and Table 3. Histone H3.1 and AFM_1_ interacted with hydrogen bonding via amino acid Thr59 and hydrophobic interactions via amino acids Val62, Leu63 and Leu97. This interaction occurred with a binding energy of − 5.30 kcal/mol and an inhibition constant of 131.29 µM. In the interaction between Histone H4 and AFM1, hydrogen-bonding interactions and hydrophobic interactions occurred with a binding energy of − 5.43 kcal/mol and inhibition constant of 105.48 µM. Histone H2A and AFM_1_ interacted by hydrogen bonds with amino acid residues Arg72 and Gln76 and hydrophobic interactions with amino acid residues Arg72, Ala75, Leu82, Arg83 and Phe84. AFM_1_ interacted with Histone H2B type 1-A by hydrogen bonding with Lys59 and hydrophobic interaction with amino acid residues Ile26, Leu62, Lys59, Ile66, Ile29 and Leu58.

**Table 3:** Potential molecular interactions and binding affinities of AFM_1_ with histone proteins.

Macromolecule	Free energy of binding (kcal/mol)	Inhibition constant (Ki)	Hydrogen bond interactions	Hydrophobic interactions
Histone H3.1	− 5.30	131.29 µM	THR59 (×2)	VAL62 (×4), LEU63, LEU97
Histone H4	− 5.43	105.48 µM	PHE70, VAL66	ILE69, ILE73 (×4), ALA110, ILE94, VAL98, PHE70
Histone H2A	− 6.92	8.44 µM	ARG72 (×2), GLN76	ARG72, ALA75, LEU82, ARG83, PHE84
Histone H2B type 1-A	− 5.05	198.56 µM	LYS59 (×2)	ILE26, LEU62 (×3), LYS59 (×2), ILE66, ILE29, LEU58

**Figure 7:**
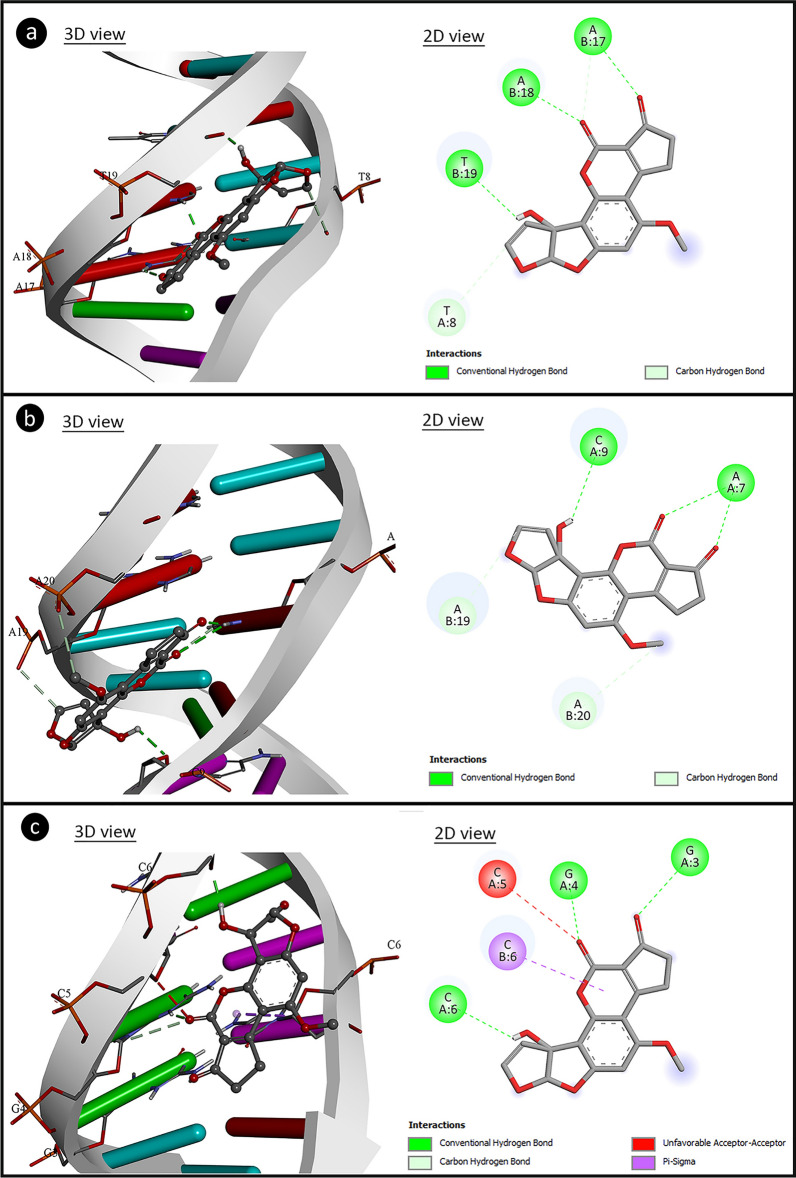
The potential molecular interactions of AFM_1_ with DNA molecules (**a**: 1BNA, **b**: 195D, **c**: 1CP8).

AFM_1_-DNA interactions were investigated by molecular docking and spectral measurements. Molecular docking of AFM_1_-DNA and the binding constants are given in Fig. 7 and Table 4. AFM_1_ had contact with B-DNA dodecamer (1BNA) with − 8.08 kcal/mol binding energy and an inhibition constant of 1.20 µM. AFM_1_ exhibited hydrogen bond interactions with T8 base in the chain A and A17, A18 and T19 in chain B. AFM_1_ formed hydrogen bonding interactions with bases A7 and C9 in chain A and with bases A19 and A20 in chain B of B-DNA Dodecamer D (195D) with binding energy − 7.54 kcal/mol and inhibiting constant of 2.97 µM. An interaction occurred between DNA (1CP8) and AFM_1_ with a binding energy of − 7.18 kcal/mol and an inhibition constant of 5.44 µM. Hydrogen bond interactions occurred between AFM_1_ and the G3, G4, C5 and C6 bases of chain A and hydrophobic interactions with the C6 base of the chain B. The results of molecular docking studies involving AFM_1_ and DNA molecules revealed that AFM_1_ has the ability to connect with DNA molecules, particularly at nucleotides in the same strand. The binding of AFM_1_ to A-A-T, A-C, A-A, G-G-C-C-C-C nucleotides can cause conformational changes in the structure of DNA. DNA-AFM_1_ molecular docking also indicates the intercalation potential of AFM_1_. Intercalation causes reductions in helix winding of DNA conformation and alters the supercoiling structure. Intercalator-induced helix relaxation can cause DNA conformational changes, DNA bending and disruption of its integrity. This conformational change on DNA is not only localized at the intercalation sites but can also proceed along the DNA chain. Intercalating agents can also cause single-strand breaks in DNA, which can occur at the intercalation point or a forward point. These breaks usually occur in the form of protein-associated DNA breaks. These proteins can be DNA repair enzymes or DNAase enzymes that cause breaks in DNA. Intercalators also have mutagenic properties and can cause many gene/chromosome mutations as well as break formations^68^. AFM_1_'s intercalator feature can cause structural changes in DNA, disruption of helical structure and integrity. This intercalator potential of AFM_1_ may also explain the formation of CAs.

**Table 4:** The binding energy of AFM_1_ with DNA molecules and interactions with nucleotides.

DNA molecule	DNA sequence	Free energy of binding (kcal/mol)	Inhibition constant (Ki)	Interacting nucleic acids *(Chain:nucleotid)*
B-DNA dodecamer (1BNA)	5′-CGCGAATTCGCG-3′	− 8.08	1.20 µM	A:T8, B:A17 B:A18, B:T19
B-DNA dodecamer D (195D)	5′-CGCGTTAACGCG-3′	− 7.54	2.97 µM	A:A7, A:C9 B:A19, B:A20
DNA (1CP8)	5′-TTGGCCAA-3′	− 7.18	5.44 µM	A:G3, A:G4 A:C5, A:C6 B:C6

**Figure 8:**
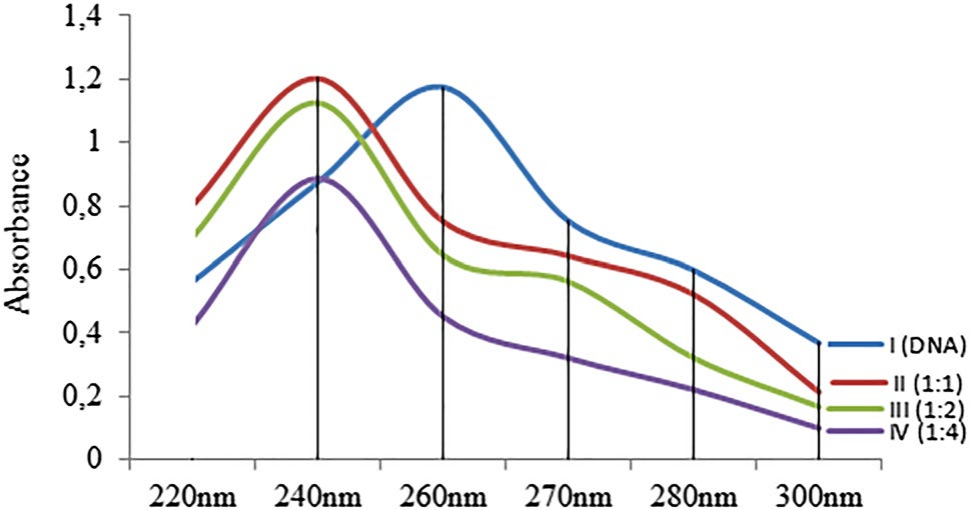
UV absorption spectrum of DNA and AFM_1_-DNA complex.

Aflatoxins exhibit genotoxic effects by many mechanisms. In addition to an indirect genotoxic effect by inducing the formation of oxidative stress, they can also create a direct genotoxic effect by interacting with DNA. The interactions of AFB_1_ with different DNA sequences and histone fractions have been investigated by various methods in the literature. It has been reported in the literature that AFB_1_ interacts with histone F2b and histone F_1_^69^. Stark et al.^70^ and Loechhler et al.^71^ reported that AFB_1_ binds to DNA and acts as an intercalator. In this study, it was determined that AFM_1_ interacts with histone H3.1, H4, H2a and H2b fractions by forming hydrogen bonds and hydrophobic interactions. In addition, AFM_1_ showed the ability to bind with DNA sequences, especially through nucleotides in the same chain. Briefly, AFM_1_ exhibited a similar mechanism to AFB_1_, interacting with the tested DNA sequences and histone proteins, and acting as an intercalator. The interaction of AFM_1_ and DNA, shown by molecular docking, was also supported by the UV absorption spectrum and the results are given in Fig. 8. The addition of AFM_1_ to the DNA solution caused hypsochromic and hyperchromic shifts in the UV spectrum. The hypsochromic shift was from 260 nm to approximately 240 nm, and the hyperchromic shift was from 1.176 to 1.202. As the AFM_1_ ratio increased, the shift intensity also increased. The hypsochromic shift confirms the AFM_1_-DNA interaction. Spectral hyperchromicity of DNA (increase in absorbance) also indicates partial instability of the secondary structure of DNA resulting from the interaction^72,73^. Molecular docking and spectral analyzes indicate the interaction of AFM_1_ with DNA. All these interactions show that the high frequency of CAs and MN formations resulting from AFM_1_ application may result from interaction with DNA and the mechanism of genotoxicity can be explained by this interaction. DNA-AFM_1_ interaction can lead to disruptions in DNA integrity, chain breaks, MN and CAs formations. Similarly, Black and Jirgensons^69^ reported that AFB_1_ binds to histone proteins and DNA, increases DNA viscosity, and causes a change in the stability of the nucleoprotein complex.

### Analysis of cytotoxic effects

**Figure 9:**
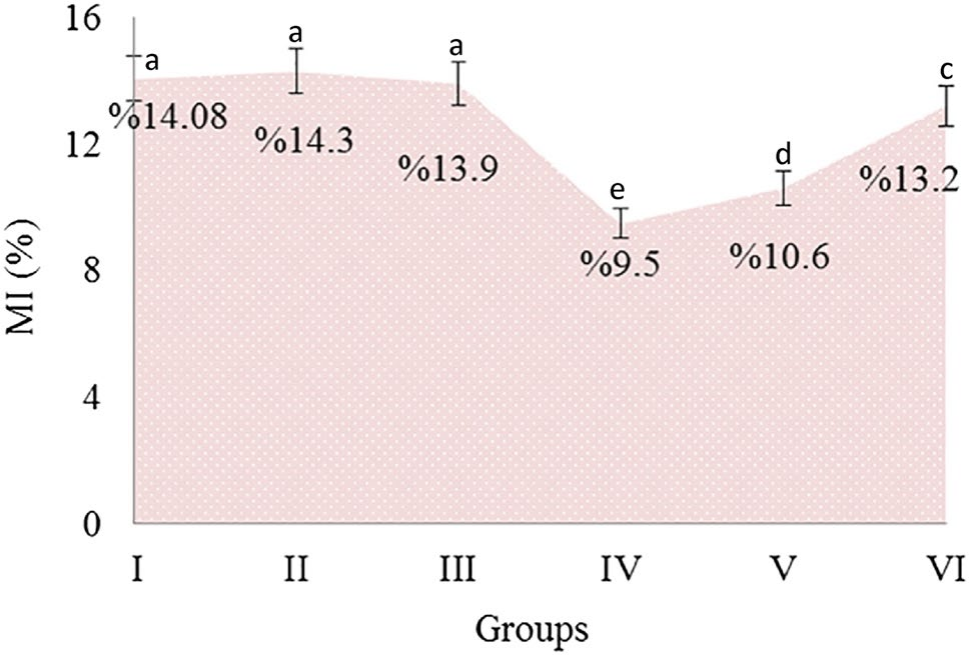
The effects of AFM_1_ and t-rsv applications on MI. MI was calculated by analyzing 1000 cells per animal (for a total of 6000 cells per group). Different letters^(a–h)^ indicated averages p < 0.05 are significant.

The effects of AFM_1_ and t-rsv on MI, which is an indicator of cell proliferation, are given in Fig. 9. MI levels in the control group, 10 mg/kg, 20 mg/kg t-rsv-treated groups were in the range of 13.9–14.3% and there was no statistical difference (p > 0.05). AFM_1_ application caused a decrease in MI rate by reducing the number of dividing cells. 16 mg/kg AFM_1_ application caused a decrease in MI rate of 32.5% compared to the control group. It was determined that MI rates improved in Group V and Group VI, which were treated with t-rsv + AFM_1_. This improvement was especially more pronounced in Group VI administered with 20 mg/kg t-rsv + AFM_1_, and MI increased 38.9% compared to Group IV treated with only AFM_1_. This healing feature of t-rsv can be explained by its regulatory role on the cell cycle. Macar et al.^74^ reported that resveratrol administration has an increasing effect on the deteriorated MI in frequently dividing meristematic cells.

### Molecular docking of AFM_1_ and tubulins

**Figure 10:**
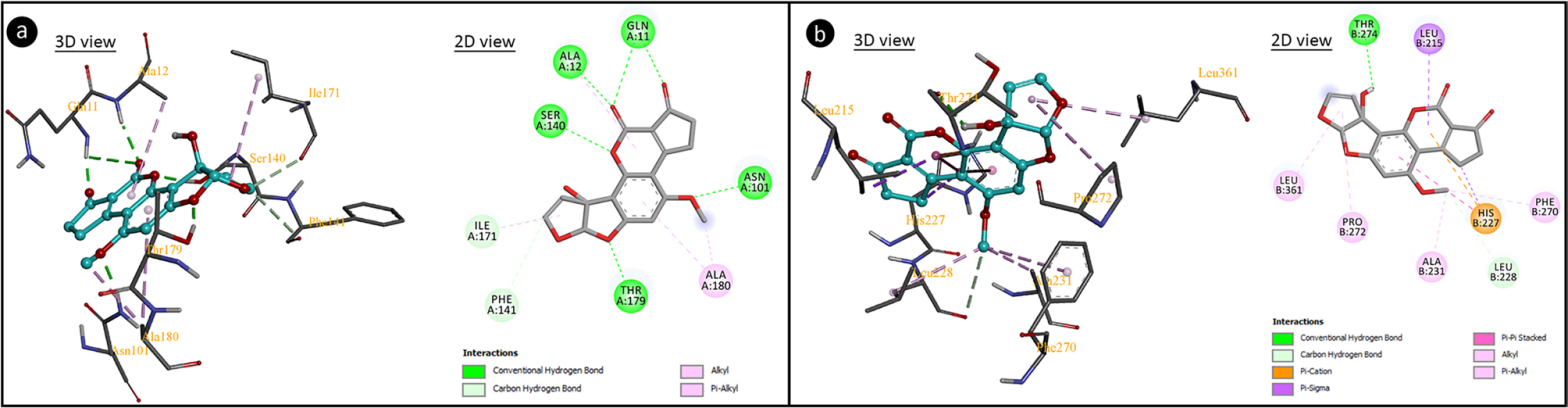
The molecular docking of AFM_1_ with tubulin proteins (**a**: α- tubulin, **b**: β- tubulin).

AFM_1_ application caused a decrease in MI rates by reducing cell proliferation in bone marrow cells. These reductions are associated with the cytotoxic effects of AFM_1_. The aneugenic effect of AFM_1_ causes spindle damage, delays and disruptions in mitosis, and these delays reduce MI rates. AFM_1_ can exhibit cytotoxic effects in many ways. In particular, the induction of large-scale MN formations by AFM_1_ (Fig. 4), indicating an aneugenic effect, suggests possible damage to the spindle fibers. From this point, tubulin proteins in the structure of spindle fibers and AFM_1_ docking were examined and the results are given in Fig. 10 and Table 5. AFM_1_ formed hydrogen bondings with the Gln11, Ala12, Asn101, Ser140, Thr179, Phe141 and Ile171 residues of the tubulin alpha-1B chain, as well as hydrophobic interactions with the Ala180, Ile171 and Ala12 residues. AFM_1_ formed hydrogen bondings and hydrophobic interactions with different aminoacid residues of tubulin beta chain with a binding energy of − 7.08 kcal/mol and an inhibition constant of 6.44 µM. The interaction between AFM_1_ and tubulin proteins can cause conformational changes in protein structure and loss of function. The γ-,α-,β-tubulin heterodimers polymerize to form microtubules and the microtubules form the spindle. The spindle apparatus, consisting of hundreds of proteins, functions in the separation of sister chromatids during cell division^75,76^. Potential AFM_1_-tubulin interaction detected by molecular docking can cause disruption of the three-dimensional structure and inhibition of microtubule polymerization. This inhibition restricts the movement of chromosomes to the poles, resulting in both disruption in mitosis and formation of CA and MN. Although there is no data in the literature on AFM1-spindle interaction, many mycotoxins have been shown to cause abnormal spindle morphology and failure of spindle formation^77^.

**Table 5:** Potential molecular interactions and binding affinities of AFM_1_ with tubulin proteins.

Macromolecule	Free energy of binding (kcal/mol	Inhibition constant (Ki)	Hydrogen bond interactions	Hydrophobic interactions
Tubulin alpha-1B chain	− 7.94	1.52 µM	GLN11 (×2), ALA12,ASN101, SER140, THR179, PHE141	ALA180 (×2), ILE171, ALA12,
Tubulin beta chain	− 7.08	6.44 µM	THR274, LEU228	LEU215, HIS227 (×3), ALA231, PRO272, LEU361, LEU228, PHE270

### Recovery effects of t-rsv

**Figure 11:**
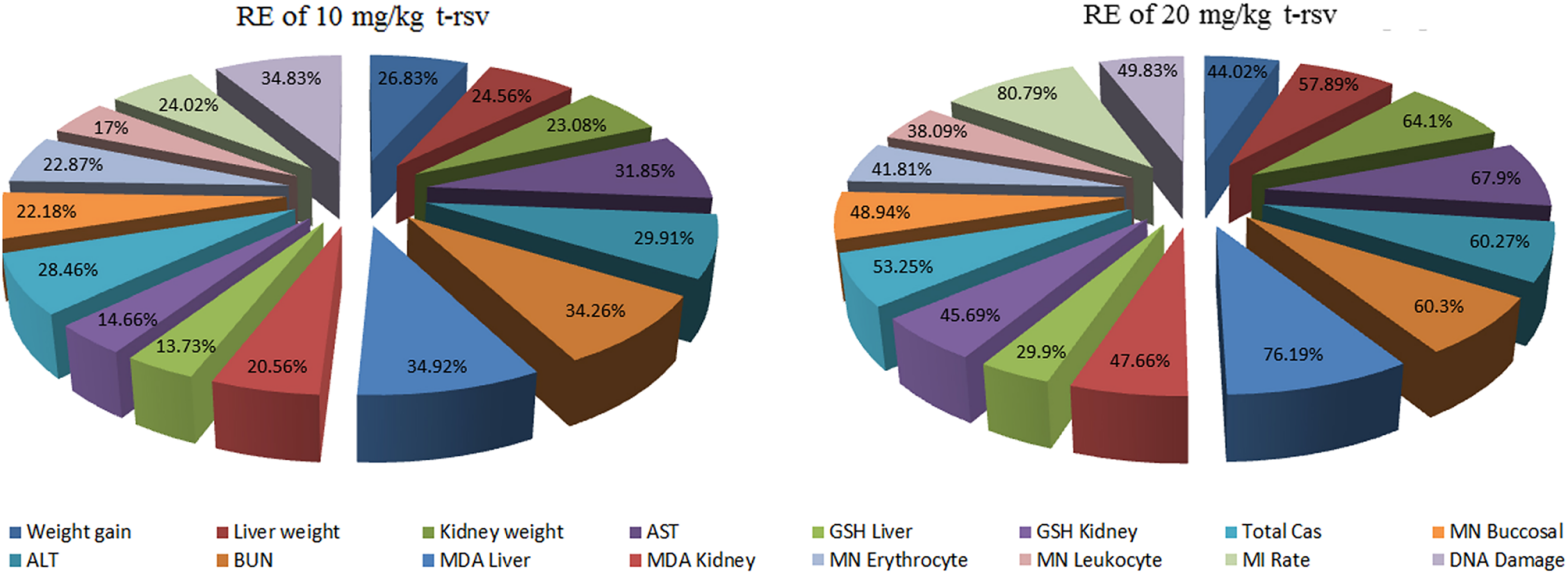
Recovery effects of 10 mg/kg and 20 mg/kg t-rsv on all parameters.

The reducing effects of t-rsv on the toxicity induced by AFM_1_ are summarized in Fig. 11. The toxicity-reducing effect of t-rsv increased in parallel with the dose increase. 10 mg/kg t-rsv and 20 mg/kg t-rsv provided protection against abnormalities in serum parameters in the range of 14.66–34.26% and 45.69–60.3%, respectively. The genotoxic and cytotoxic effects of AFM_1_ were determined by examining the MI rate, formation MN and CAs, and 20 mg/kg t-rsv reduced the frequency of MN by 48.9% and improved the rate of MI by 80.7%. 10 mg/kg and 20 mg/kg t-rsv treatment with AFM_1_ resulted in a 22.6–63.9% reduction in CAs types. The most significant reduction was observed in gap formation, and 20 mg/kg t-rsv application reduced gap formation by 63.9%. There are many studies in the literature on the protective properties of resveratrol. However, in some studies, it is reported that resveratrol is cytotoxic^78^, and in some studies it has no toxic effect even at high doses^79^. In this study, 10 mg/kg and 20 mg/kg doses of resveratrol were tested, and it was determined that it did not have a toxic effect at these doses and showed a protective feature against AFM_1_ toxicity.

### Correlation and principal component analysis of parameters

**Figure 12:**
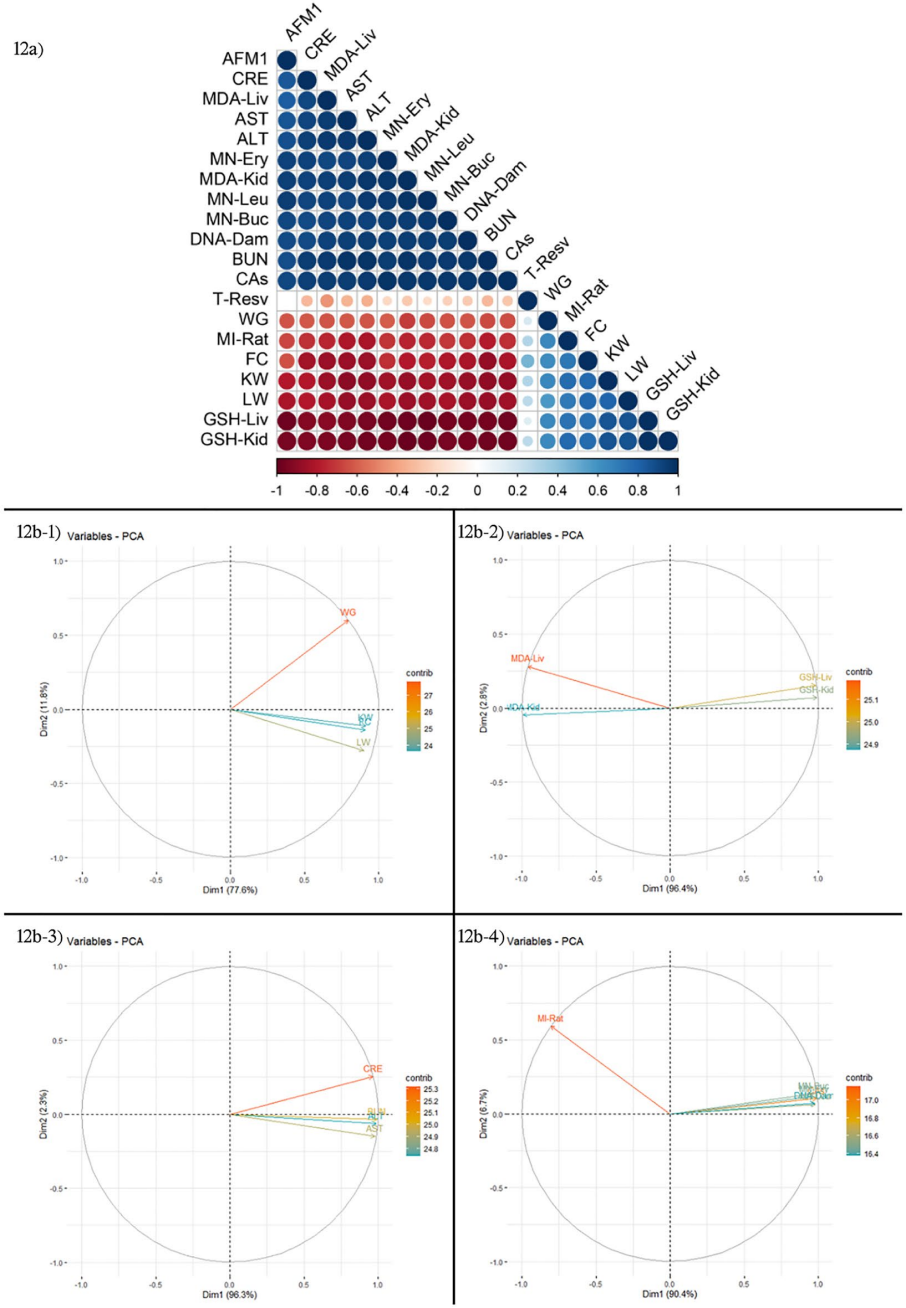
(**a**) Correlation of AFM_1_ and t-resv with physiological, biochemical and genetic parameters. Pearson correlation analysis (two-sided) was performed and visualized with Rstudio software. Positive correlations are shown in blue and negative correlations in red. The color intensity and the size of the circle are proportional to the correlation coefficients, (**b**) Principal Component Analyses (PCA) of physiological, biochemical and genetic response parameters.

Figure 12a shows the correlation analysis of all parameters. Positive correlations are denoted by the color blue, whereas negative correlations are represented by the color red. Correlation coefficients are related to the color intensity and circle size. CAs, DNA Damage, buccal mucosa MN, erythrocyte MN, leukocyte MN, MDA liver, MDA kidney, ALT, AST, BUN and creatinine levels all showed a positive correlation with AFM_1_, but weight gain, MI rate, feed consumption, kidney weight, liver weight, GSH liver and GSH kidney levels all showed a negative correlation. T-resv was shown to have a positive correlation with weight gain, MI rate, feed consumption, kidney weight, liver weight, GSH liver and GSH kidney levels, but a negative correlation with CAs, DNA damage, buccal mucosa MN, erythrocyte MN, leukocyte MN, MDA liver, MDA kidney, ALT, AST, BUN, and creatinine levels, showing that it has a protective impact.

Principal component analysis (PCA) was used to visualize the overall physiological, biochemical, and genetic impacts of AFM1 and t-rsv treatments on Swiss albino mice, as well as clustering amongst biomarkers after the application period and are given in Fig. 12b. PCA was used to visualize the overall physiological, biochemical, and genetic impacts of AFM1 and t-rsv treatments on Swiss albino mice, as well as clustering amongst biomarkers after the application period. Analysis of different toxicity biomarkers has provided a more reliable and more comprehensive view of the toxicity status and the interrelationships of these parameters. To minimize the complexity of data interpretation of multiple biomarker analysis, the current research used the statistical data-reduction tool PCA. In the current study, PCA analyzes of 4 physiological, 4 organ biochemistry, 4 blood biochemistry and 6 genetic parameters were analyzed and their relationships were examined. In Fig. 12b-1, which deals with PCA analyzes of physiological parameters, the first two dimensions of the biplot explained 89.4% of the overall variance, with the first axis (dim1) distinguishing control and treatment groups clearly (77.6%). The dim2 as a visualization aid accounted for 11.8% of the overall variance. As a result of the analysis, it was found that kidney weight, liver weight and feed consumption levels were close to each other, with a very positive component on the dim1 axis and a slight negative on the dim2 axis. Weight gain was on the positive axis of dim2 and dim1. PCA analyzes of organ biochemistry parameters are given in Fig. 12b-2. In the biplot, the first two dimensions, the first axis (dim1) 96.4% and the second axis (dim2) 2.8%, explain 99.2% of the overall variance. As a result of the analysis, it was found that GSH liver and GSH kidney levels were close to each other, with a very positive component on the dim1 axis and a slight positive on the dim2 axis. MDA liver and MDA kidney levels were found to be very negative on the dim1 axis and slightly positive on the dim2 axis. MDA liver levels were moderately positive in the dim2 axis, while MDA kidney levels were mildly negative. Fig. 12b-3 shows PCA analyses of blood biochemistry parameters. The first two dimensions in the biplot, the first axis (dim1) 96.3% and the second axis (dim2) 2.3%, explain 98.6% total variation. As an outcome of the assessment, it was found that creatinine, ALT, AST and BUN levels were very positive components on dim1 axis. It was determined that the levels of ALT, AST and BUN levels were close to each other, slightly negative in the dim2 axis and creatinine level was a slightly positive component in the dim2 axis. PCA analyzes of genetic parameters are given in Fig. 12b-4. The first two dimensions in the biplot, the first axis (dim1) 90.4% and the second axis (dim2) 6.7%, explain 97.1% total variance. As a result of the analysis, it was observed that the MI ratio had a very negative component in the dim1 axis and a positive component above the moderate level in the dim 2 axis. Buccal mucosa MN, erythrocyte MN, leukocyte MN, CAs and DNA Damage frequencies were found to be very positive components and very close to each other in the dim1 axis. These parameters are on the positive side of the dim2 axis and close to the axis. All these PCA analyzes confirm the interrelationships of the investigated parameters with each other.

## Conclusion

Among mycotoxins, aflatoxins are the most effective on higher organisms, and it is very difficult to classify the effects of aflatoxins clearly since direct studies on humans cannot be conducted. Although there are many studies on aflatoxin species with chronic and acute effects in animals, studies on AFM_1_ are generally related to the detection of its presence in milk and dairy products. In this study, the potentially toxic effects of AFM_1_ in albino mice and the toxicity limiting property of t-rsv against these toxic effects were investigated. AFM_1_, which causes significant changes in selected physiological parameters, liver and kidney markers in albino mice, is an agent with cytotoxic and genotoxic effects and disrupts the antioxidant/oxidant balance. AFM_1_ exhibited cytotoxic and genotoxic effects, respectively, by interacting with tubulin proteins, which are involved in cell division, and histone proteins, which have an important role in packaging and maintaining the integrity of DNA. With this study, the first data entry was provided to the literature regarding the formation MN in the buccal epithelium, leukocyte and erythrocyte induced by AFM_1_.

There are much data in the literature regarding the protective properties of resveratrol, which has antioxidant properties. However, there is no study reporting the protective effect of t-rsv against toxicity induced by AFM_1_. In this regard, this study is the first to report that t-rsv exhibits a dose-dependent protective role against AFM_1_ toxicity. In the sustainability of high quality of life, it is very important to clarify the toxic effects of chemicals that contaminate organisms and to conduct research to reduce these effects. Studies investigating the toxic effects of chemicals and the toxicity-reducing role of natural products against these effects and elucidating the mechanism of toxicity are very valuable. From this point of view, this study will guide many studies examining the mechanisms of toxicity and the toxic-reducing role of natural compounds.

## Data Availability

The datasets used and/or analyzed during the current study are available from the corresponding author on reasonable request.
